# Identification of placental genes linked to selective intrauterine growth restriction (IUGR) in dichorionic twin pregnancies: gene expression profiling study

**DOI:** 10.1007/s00439-019-02016-6

**Published:** 2019-04-30

**Authors:** Lidia Biesiada, Agata Sakowicz, Mariusz Grzesiak, Maciej Borowiec, Michalina Lisowska, Tadeusz Pietrucha, Constantin von Kaisenberg, Krzysztof Lewandowski

**Affiliations:** 1 Department of Obstetrics, Perinatology and Gynecology, Polish Mother’s Memorial Hospital-Research Institute in Lodz, Rzgowska 281/289, Lodz, Poland; 2 Department of Medical Biotechnology, Medical University of Lodz, Lodz, Poland; 3 Department of Clinical Genetic, Medical University of Lodz, Lodz, Poland; 4 Department of Obstetrics and Gynecology, Hannover Medical School, Hannover, Germany; 5 Department of Endocrinology and Metabolic Diseases, Polish Mother’s Memorial Hospital-Research Institute in Lodz, Lodz, Poland

## Abstract

A linkage of dichorionic (DC) twin pregnancies with selective intrauterine growth restriction (IUGR) to alterations in placental gene expression is unclear. The aim of the study was to identify placental genes related to hypoxia, adipogenesis and human growth which may contribute to IUGR development. The study group (IUGR/AGA) comprised dichorionic (DC) twin pregnancies, where the weight of the twins differed by > 15%; in addition, one twin was small for gestational age (< 10th percentile-SGA) (IUGR) while the other was appropriate for gestational age (> 10th percentile-AGA). In the control group (AGA/AGA), both fetuses were AGA and their weights differed by < 15%. In the first step (selection), placental expression of 260 genes was analysed by commercial PCR profiler array or qPCR primer assay between six pairs of IUGR/AGA twins. In the second stage (verification), the expression of 20 genes with fold change (FC) > 1.5 selected from the first stage was investigated for 75 DC pregnancies: 23 IUGR/AGA *vs.* 52 AGA/AGA. The expression of Angiopoetin 2, Leptin and Kruppel-like factor 4 was significantly higher, and Glis Family Zinc Finger 3 was lower, in placentas of SGA fetuses (FC = 3.3; 4.4; 1.6; and − 1.8, respectively; *p* < 0.05). The dysregulation of gene expression related to angiogenesis and growth factors in placentas of twins born from IUGR/AGA pregnancies suggest that these alternations might represent biological fetal adaptation to the uteral condition. Moreover, DC twin pregnancies may be a good model to identify the differences in placental gene expression between SGA and AGA fetuses.

## Introduction

Intrauterine growth restriction (IUGR) is an important cause of fetal and neonatal morbidity and mortality. As it is usually defined for singleton pregnancies as the rate of fetal growth below the 10th percentile of a population-specific birth weight for gestational age, the terms *IUGR* and small for gestational age (SGA) are often used interchangeably (Sharma et al. 2016). For twin pregnancies, IUGR is a condition in which one or two siblings do not grow well. In addition, various criteria exist for IUGR diagnosis twin pregnancies; however, the following two are used most frequently: (1) either twin with a birth weight < 10th percentile for gestational age, (2) the presence of a 10–30% difference in birthweight between co-twins (Breathnach and Malone 2012; Moore and O’Brien 2006; Ropacka-Lesiak et al. 2012; Yinon et al. 2005).

It is now generally accepted that IUGR is related to abnormal placentation, resulting in malnutrition and reduced oxygen supply to the fetus. Primary hypoxia is required at the very early stages of blastocyst implantation for appropriate placentation to take place, as it stimulates cytotrophoblast proliferation and angiogenesis (Khalig et al. 1999; Wallner et al. 2007). However, a prolonged reduction of oxygen availability results in restricted fetal growth (Ream et al. 2008) and may affect the expression of many adipogenesis genes (Díaz et al. 2013; Sagawa et al. 2002). Alternations in placental gene expression profile associated with hypoxia may not only play a key role in the development of IUGR, but may also influence the programming of metabolic disorders occurring in postnatal life (Struwe et al. 2010).

Observations of twin pregnancies, i.e. where both fetuses had been developing in the same maternal environment and at the same time, indicate that the local environment created by the area of implantation in the decidua was the only factor differentiating the two fetuses (Chang et al. 2013). This inequality in placental territory may influence fetal development and differentiate their growth (Chang et al. 2013). As the disparity in fetal growth between a pair of twins in the same mother cannot be attributed to confounding maternal variables (Westwood et al. 2001), the best control for the SGA fetus seems to be its AGA co-twin.

We believe that dichorionic (DC) twin pregnancy is a better model for separating the influence of local factors (uterine and placental) from maternal homeostasis factors than monochorionic (MC) twin pregnancy. This model eliminates the effect of anastomoses between siblings: 95% of MC twins possess vascular anastomoses on the placental surface, their presence slows the development of one of the fetuses by reducing the blood supply (Lewi et al. 2013). In contrast, placental anastomoses appear only occasionally in DC gestation.

To check the impact of impaired placentation on fetal development, the study compares the expression of selected genes by SGA fetuses and their AGA co-twins from IUGR pregnancies. We hypothesize that the SGA and AGA siblings of the IUGR/AGA pregnancy may differ in expression of placental genes engaged in adipogenesis, hypoxia and human growth may differ between them. Any difference in the gene expression may be related with the adaptation of the fetuses to the intrauterine environment.

Moreover, the present study compares the levels of gene expression between twins born from IUGR/AGA and AGA/AGA pregnancies to determine whether maternal homeostasis may influence the modulation of mRNA level by the fetus. The study assumes that when a difference in gene expression is observed only between the AGA and SGA co-twins born from a IUGR/AGA pregnancy, this expression is strictly linked with the local uterine environment created by placentation; however, when such a difference is observed between twins born from IUGR/AGA and AGA/AGA pregnancies, these genes are generally regulated by maternal homeostasis.

## Materials and methods

**Fig. 1:**
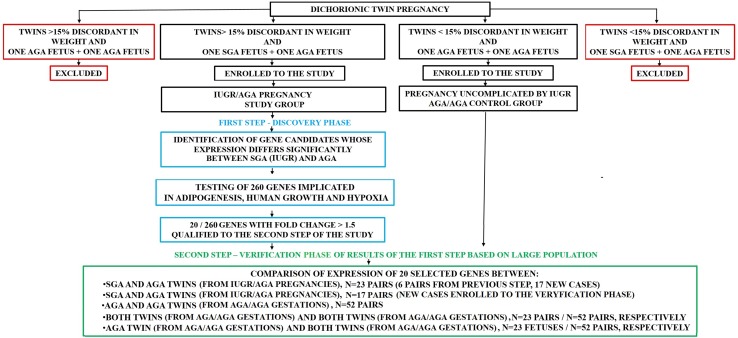
Algorithm of the study. *SGA* fetus delivered with the small weight for gestational age that means with the weight lower than the 10th percentile for the gestational age, *AGA* fetus delivered with the weight appropriate for the gestational age that means with the weight between the 10th and 95th percentile for the gestational age

The whole study was conducted according to scheme presented by the Fig. 1.

### Clinical characterization of subject

The study included 75 DC twin pregnancies: 23 pregnancies with IUGR/AGA, defined as fetus birth weight discordance ≥ 15% and the weight of one sibling below 10th percentile whereas the weight of its co-twin was between the 10th and the 95th percentile. Control group consists of 52 twin AGA/AGA pregnancies whose weight difference below 15% and the weight of both siblings was between the 10th and 95th percentile. The exclusion criteria for IUGR/AGA and AGA/AGA groups comprised the presence of chromosomal abnormalities, placental vascular abnormalities and infarction. Premature rupture of membranes (PROM), pregnancy-induced hypertension, maternal hypothyroidisms and gestational diabetes mellitus did not exclude the patients from the study. Both groups included similar proportions of women who had conceived by IVF, delivered twins by Caesarian section or vaginally, and both groups included similar proportions of fetuses with regard to sex, i.e. male–male, female–female, male–female. Dichorionic twin pregnancy was identified based on ultrasonography conducted in the first trimester of gestation.

All patients were recruited in the Polish Mother’s Memorial Hospital Research Institute, Lodz, Poland. All clinical investigations were conducted in accordance with the guidelines of The Declaration of Helsinki, and approved by the Ethical Committee of the Polish Mother’s Memorial Hospital Research Institute (No 85/2013). All patients gave informed consent before joining the study.

### Laboratory examinations

Data concerning maternal age and pre-pregnancy weight were provided by participants. Body mass index (BMI) was calculated by dividing the weight of the participant in kilograms (kg) by height in meters (m) squared. Maternal weight growth per week (kg) was calculated based on information taken from the pregnancy chart. Maternal blood samples were collected from the patients before delivery. The following haematological tests were performed according to standard laboratory methods in the diagnostic laboratory of the hospital: white blood count (WBC), red blood count (RBC), haemoglobin level (Hb), Haematocrit (Ht), mean corpuscular volume (MCV), corpuscular/cellular haemoglobin concentration (MCHC).

### Biopsy procedure and RNA isolation

Immediately after the birth of the fetuses, a 5 cm-long fragments of each placenta were taken from the site of the umbilical cord attachment. The amniotic membranes and maternal decidua were removed. The placenta sample was washed in PBS and placed in the tube with RNA later.

Total RNA was isolated using a commercial Rneasy Mini Kit (Qiagen, Germany) according to the manufacturer’s protocol. The concentration and purity of RNA were measured by NanoDrop (Thermo Fisher, USA). The 280/260 absorbance ratio for the RNA sample was found to be between 1.8 and 2.1, indicating that the sample was of sufficient quality for further analysis.

### Reverse transcription and PCR arrays in discovery phase

An RNA sample (1 µg) was used for the reverse transcriptase reaction (RT^2^ SYBR^®^ Green qPCR Mastermix, Qiagen, USA). The resulting cDNA was diluted fivefold in DNA- and RNA-free distilled water and stored at − 20 °C until use.

Three commercial RT2 Profiler PCR Arrays (Qiagen, USA) were used to determine the expression profile of 254 genes in each placenta sample taken from six pairs of twins born from IUGR/AGA pregnancies. The first of the arrays included 84 genes related to growth factors, the second included 84 involved in adipogenesis and the third included 84 genes related to the response to low oxygen level (hypoxia). Gene expression array studies were performed according to the manufacturer’s instructions in a LightCycler 480 (Roche) device. As well as the 84 studied genes, the PCR array also contained the following 12 internal control genes: five housekeeping genes (beta-2-microglobulin (*B2* *M)*, *h*ypoxanthine phosphoribosyltransferase 1 (*HPRT1*), Ribosomal Protein Lateral Stalk Subunit P0 (*RPLPO*), Glyceraldehyde-3-Phosphate Dehydrogenase (*GAPDH*) and Actin Beta (*ACTB*)), three reverse transcription controls, three positive Real-time PCR controls and one human genomic DNA contamination control (HGDC). The results were normalised according to the mean of the housekeeping genes, for which the expression stability was in the range of 0.016–0.063, as indicated by the NormFinder tool (Andersen et al. 2004). The relative expression of each gene was determined by the fold change (2^−ΔΔCt^) method, as described elsewhere (Demes et al. 2012; Gao et al. 2013; Livak and Schmittgen 2001). A fold change ≥ 1.5 indicated a significant change in gene expression.

The three Real-time PCR control genes (PPC) and three reverse transcription genes (RTC) were used as internal controls of reaction and for calculation of inter-plate variability; the calculation of coefficient of variability (CV) for each type of plate was described elsewhere (Dunk et al. 2000; Tavano et al. 2017). The coefficient of variability calculated for all arrays together was as follows (CV_RTC_ = 14%; CV_PPC_ = 9%). Intra-assay variations are in an acceptable range for CV < 25% (Demes et al. 2012).

This step analysed the expression profiles of six additional genes related to genetic disorders characterised by the low fetal weight: *IGFR2*, *GLIS3*, *MAGEL*, *H19*, *GRB10,* and *PHLDA2*. These were included into the analysis using a commercial primer assay (RT2 qPCR Primer Assay, Qiagen, USA). The relative expression of these genes was calculated by the Pfaffl method (Pfaffl 2002).

At the end of the first step the twenty genes were selected based on the fold change > ±1.5 between IUGR (SGA) and AGA co-twins. The list of selected genes was presented in the Table 1.

**Table 1:** Genes investigated in the second step of the study

Unigene	GeneBank	Symbol	The full name of the gene
Adipogenesis pathway			
Hs.583870	NM_001147	*ANGPT2*	Angiopoietin 2
Hs.591251	NM_000024	*ADRB2*	Adrenergic, beta-2-, receptor, surface
Hs.473163	NM_001719	*BMP7*	Bone morphogenetic protein 7
Hs.699463	NM_004364	*CEBPA*	CCAAT/enhancer-binding protein (C/EBP), alpha
Hs.202354	NM_000793	*DIO2*	Deiodinase, iodothyronine, type II
Hs.391561	NM_001442	*FABP4*	Fatty acid-binding protein 4, adipocyte
Hs.367725	NM_032638	*GATA2*	GATA binding protein 2
Hs.465744	NM_000208	*INSR*	Insulin receptor
Hs.376206	NM_004235	*KLF4*	Kruppel-like factor 4 (gut)
Hs.194236	NM_000230	*LEP*	Leptin
Hs.696032	NM_006238	*PPARD*	Peroxisome proliferator-activated receptor delta
Hs.466693	NM_012237	*SIRT2*	Sirtuin 2
Growth factors			
Hs.40499	NM_012242	*DKK1*	Dickkopf homolog 1 (Xenopus laevis)
Hypoxia			
Hs.523012	NM_019058	*DDIT4*	DNA-damage-inducible transcript 4
Hs.514505	NM_015167	*JMJD6*	Jumonji domain containing 6
Hs.707978	NM_000603	*NOS3*	Nitric oxide synthase 3 (endothelial cell)
Hs.2795	NM_005566	*LDHA*	Lactate dehydrogenase A
Hs.372914	NM_006096	*NDRG1*	N-myc downstream regulated 1
Others genes related to growth			
Hs.533566	NR_002196	*H19*	H19, imprinted maternally expressed transcript (non-protein coding)
Hs.162125	NM_001042413	*GLIS3*	GLIS family zinc finger 3

### Quantitative RT-PCR in validation phase

A total of 75 DC pregnancies were enrolled: 23 with IUGR/IUGR (6 pairs from the first step and 17 new pairs of twins) with birthweight discordance > 15% and one sibling with weight < 10th percentile (SGA sibling), and 52 with birth weight discordance below 15% and both siblings had weight between the 10th and 95th percentile. A custom RT2 PCR Array CLAH27161 (Qiagen, USA) was used to analyse the 20 genes selected in the first step in this population.

The relative expression of each gene was determined by the fold change (2^−ΔΔCt^) method, as described previously. The inter-assay coefficient of variability was calculated at CV = 7.7%; the acceptable range for CV is below 25%, as noted elsewhere (Demes et al. 2012).

### Statistical analysis

Statistical analyses were performed using Statistica version 13.1 (Statsoft, Poland). The distribution of the analysed haematological, clinical, and expression data was checked by the Shapiro–Wilk test. Normally distributed data were further analysed with the Student’s *t* test, while non-normally distributed data were analysed using Mann–Whitney *U* test. The *p* values for gene expression were calculated using the Mann–Whitney *U* test on 2^−ΔCt^ values calculated separately for each twin. The categorical variables were calculated by the Chi-square test or with Yates’s correction. The relative gene expression among three sets of siblings selected according to their gender (girl–girl, boy–boy, boy–girl) was evaluated using the Kruskal–Wallis test with the post hoc Dunn’s test. A *p* value below 0.05 for the test results was considered as statistically significant.

## Results

The clinical and laboratory test data, as well as pregnancy and delivery characteristics, are presented in Table 2.

No significant differences were observed between the study and control groups with regard to BMI, maternal haematological parameters or pregnancy outcomes. Maternal age was significantly higher in the study group compared to controls. No difference was found in newborns according to sex and to the week of delivery.

**Table 2:** A comparison of the clinical, haematological and pregnancy-related characteristics between IUGR/AGA pregnancies and subjects with less than 15% birth weight discordance between twins (AGA/AGA group)

Maternal parameter	IUGR/AGA group (*N* = 23 cases)	AGA/AGA group (*N* = 52 cases)	*p* value
Maternal age (years)^a^	33 ± 4.1	31 ± 4.9	< 0.009
Week of delivery^a^	35 ± 2.4	35 ± 2.1	0.697
Maternal weight growth per week (kg)^a^	0.44 ± 0.1	0.40 ± 0.1	0.114
RBC (10^6^/μl)^a^	4.1 ± 0.36	4.1 ± 0.38	0.994
Hb (g/dl)^a^	12.1 ± 1.6	12.4 ± 1.2	0.322
Ht (%)^a^	35.1 ± 3.5	36.6 ± 3.2	0.307
PLT (10^3^/μl)^a^	181 ± 53	191 ± 63	0.526
BMI (kg/m^2^)^b^	23 (19–26)	23 (21–25)	0.885
WBC (10^3^/μl)^b^	9.8 (7.5–11.8)	9.9 (8.8–12.7)	0.448
MCV (fl)^b^	87 (83–88)	87 (83–91)	0.262
MCHC (g/dl)^b^	34 (32–35)	34 (33–35)	0.836
Primiparous^c^	13 (56%)	33 (63%)	0.755
Miscarriage^c^	0 (0%)	0 (0%)	–
Assisted fertilisation (IVF)^c^	5 (22%)	14 (27%)	0.851
Caesarean section^c^	20 (87%)	48 (92%)	0.76
Cholestasis^c^	4 (17%)	5 (10%)	0.569
GDM^c^	4 (17%)	8 (15%)	0.902
HA^c^	6 (26%)	7 (13%)	0.317
Hypothyroidism^c^	4 (17%)	10 (19%)	0.894
PPROM^c^	4 (22%)	13 (28%)	0.896
Uterus contractions^c^	13 (56%)	16 (31%)	0.040
Using oxytocin during delivery^c^	6 (26%)	6 (11%)	0.214
Placenta localisation in uterus different than anterior or posterior^c^	4 (17%)	12 (23%)	0.804

Child parameters	Study group (SGA + AGA) (23 pairs of twins)	Control group (AGA) (52 pairs of cases)	*p* value
Weight of the child (g)^b^	SGA + AGA 2375 (2100-2600) where SGA 1948 (1650–2360) AGA 2500 (2120–2900)	2400 (2235–2610)	0.037*
Male newborns^c^	26 (57%)	55 (53%)	0.680**

*BMI* body mass index, *WBC* white blood cells, *RBC* red blood cells, *HB* haemoglobin concentration, *HCT* haematocrit, *MCV* mean corpuscular volume, *MCHC* mean corpuscular haemoglobin concentration, *PLT* platelets, *kg/m*^*2*^ kilograms/meter square, *µl* microlitre, *g/dl* grams/decilitre, % percent, *fl* fentolitre, *g* grams, *SGA* small to gestational age twin, *AGA* appropriate for gestational age twin, *PPROM* Preterm premature rupture of membranes, *GDM* Gestational diabetes mellitus, *HA* hypertension, *IVF*, in vitro fertilisation

^a^Data presented as mean ± standard deviation; *p* value calculated using *t* Student test

^b^Data presented as median and lower–upper quartile, *p* value calculated using *U* Mann–Witney test

^c^Data presented as the number of cases and percent (%), *p* value calculated using chi-square test

### The first stage of the study—discovery phase

Six pairs of twins from the discordant group were chosen randomly for the first step of our study. The expression profiles of 260 genes of the SGA babies were compared with those of their AGA siblings. The univariate analyses indicated that 20 genes varied between the SGA and AGA siblings (fold change > 1.5) (Table 3). These 20 genes were qualified to the next step of the study.

**Table 3:** Relative fold changes in placental gene expressions of the SGA (IUGR) twin in comparison to the AGA twin from IUGR/AGA pregnancies

Studied gene	FC*	*p* value
Adipogenesis pathway		
*ANGPT2*	20.2	0.031
*DIO2*	18.4	0.229
*LEP*	16.2	0.031
*BMP7*	9.5	0.093
*CEBPA*	4.9	0.298
*KLF4*	4.1	0.128
*INSR*	3.3	0.575
*GATA2*	2.7	0.229
*ADRB2*	2.4	0.093
*PPARD*	2.35	0.298
*FABP4*	2.3	0.575
*SIRT1*	2.2	0.471
Hypoxia pathways		
*LDHA*	− 2.8	0.229
*NDRG1*	− 2.6	0.298
*NOS3*	− 2.2	0.297
*DDIT4*	− 2.2	0.378
*JMJD3*	− 1.9	0.575
Growth factors		
*DKK1*	− 2.5	0.235
Other genes related to growth		
*H19*	− 1.8	0.575
*GLIS3*	3.6	0.378

The results of the first stage of the study based on six pairs of twins

*FC Fold change was calculated based on relative gene expression calculated by 2^−ΔΔCt^ method or Pfaffl method for *H19* and *GLIS3* genes

**p value calculated using the Mann–Whitney *U* test

### Second step of the study—verification phase

The levels of expression of twenty genes chosen in the first step were analysed in 75 twin pregnancies: 23 from study IUGR group (6 pairs from the first stage and 17 new pairs of twins) and 52 from AGA/AGA pregnancies (control group).

**Fig. 2:**
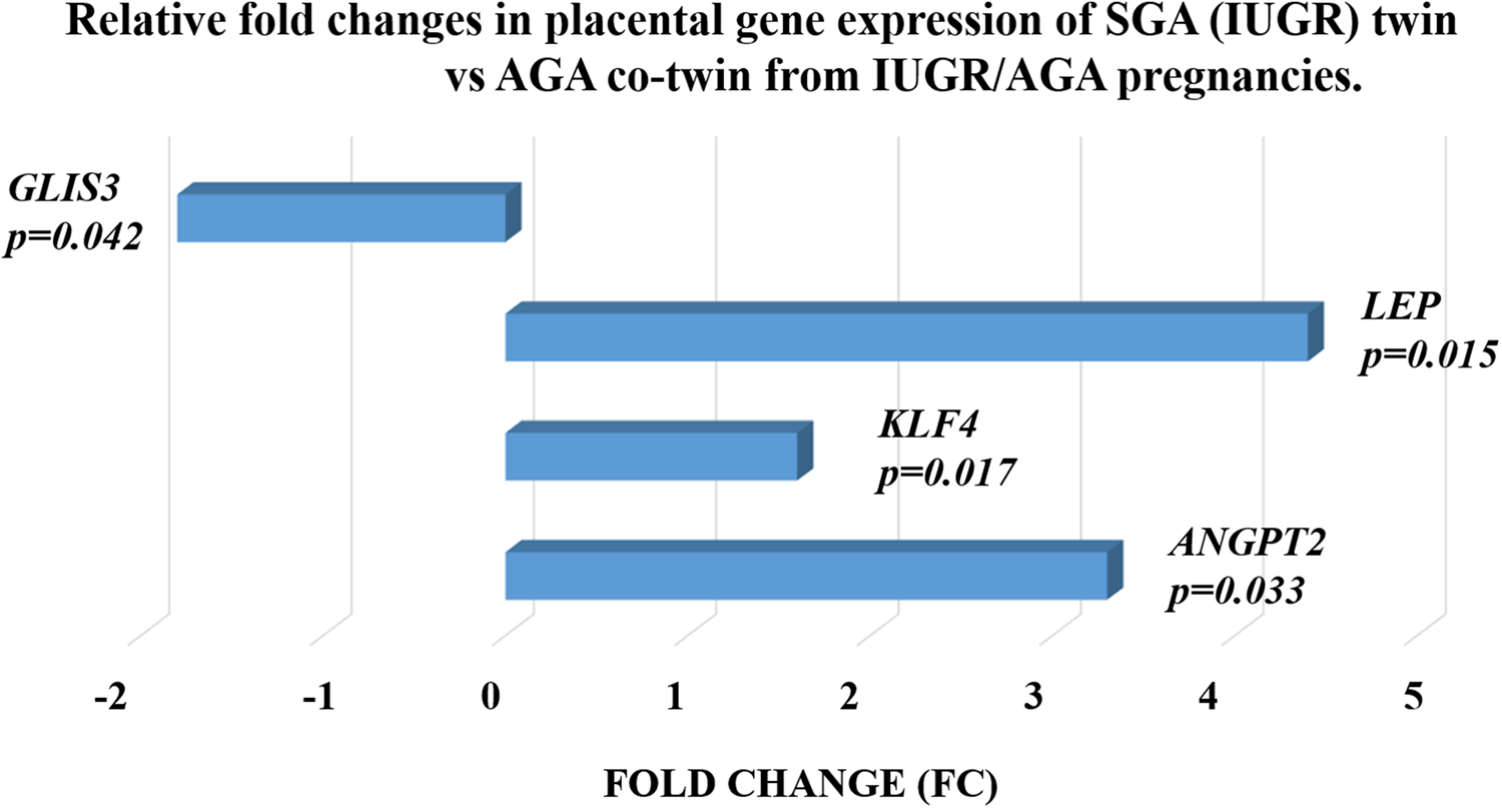
Relative fold changes in placental gene expression by the SGA twin of an IUGR pregnancy compared to its AGA co-twin. The analysis was performed on 23 pairs of twins during the second stage of the study. Fold change was calculated based on relative gene expression calculated by the 2^−ΔΔCt^ method; the *p* value was calculated using the Mann–Whitney *U* test

The initial test examined which of the chosen genes could be associated with the development of IUGR independent of maternal environment, so analyses were conducted only on fetuses from the discordant group. The univariate analyses of SGA fetuses and their AGA co-twins indicated four genes which were significantly associated with the local environment that may have influenced the IUGR development (Fig. 2). No such difference was observed between the smaller and the larger co-twins (weight discrepancy < 15%) in the AGA/AGA group.

To eliminate the influence of the results from the discovery phase on the result achieved in the verification phase, we decided to analyse the population of twins from IUGR/AGA pregnancies, but after elimination of the six pairs of twins from the validation stage. The Mann–Whitney *U* test based on the 17 new pairs of twins enrolled to the verification phase found that the occurrence of the two genes *GLIS3* and *KLF4* differs significantly between SGA and AGA twins (fold change − 1.8, fold change 1.5, respectively), whereas the expression of *LEP* and *ANGPT2* was insignificantly higher in the SGA twins than the AGA twins (fold change 2.4 and 2.0, respectively).

An analysis was carried out on the study group to determine whether the arrangement of fetuses’ sex (female–female, male–male, male–female) influences gene expression. The Kruskal–Wallis analysis indicated that none of the genes apart from *DDK1* were associated with the combination of sexes for each set of twins. However, this relationship failed the post-hoc Dunn’s test (*p* > 0.05), indicating that this gene does not play any significant role in the IUGR development.

In the next step, the IUGR (AGA and SGA together) populations of twins and the AGA/AGA populations (all twins together) were compared with regard to the relative expressions of all genes qualified to the second step. *CEBPA* and *FABP4* expression was significantly lower in the placentas obtained from the IUGR/AGA twin population (AGA and SGA together) than the AGA/AGA group (FC = − 1.7; *p* = 0.011 and FC = − 1.7; *p* = 0.008; respectively). Interestingly, a comparison of AGA twins from the IUGR/AGA pregnancies and those from AGA/AGA pregnancies (AGA + AGA) indicated that *CEBPA* and *FABP4* genes were also significantly downregulated in AGA twins from IUGR/AGA gestations (FC = − 2.3 and FC = − 2.1; *p* < 0.01; respectively). In addition, *ANGPT2* and *KLF4* gene expression was significant lower in the population of the AGA twins from IUGR/AGA gestations than those from the AGA/AGA pregnancies (FC = − 2.9 and FC = − 2.3; *p* < 0.01; respectively).

The association between the fetus sex and the gene expression was compared between the placentas from IUGR/AGA pregnancies and the AGA/AGA pregnancies. The two sexual groups demonstrated different profiles of gene dysregulation. *FABP4* and *PPARD* expression was downregulated in female placentas from the IUGR pregnancies (FC = − 2.8; *p* = 0.014 and FC = − 1.5; *p* = 0.046; respectively). In contrast, male placentas from IUGR group demonstrated downregulated *CEBPA* expression (FC = − 2.1; *p* = 0.039).

## Discussion

It is generally accepted that the common cause for IUGR development is abnormal placentation. This leads to a restriction in oxygen and nutrient supply through the placenta to the fetus thus resulting in placental hypoxia and its apoptosis (Erel et al. 2001).

The present study found significantly increased expression of *ANGPT2*, *KLF4,* and *LEP* but lower expression of *GLIS3* gene in the placentas of SGA twins compared to their AGA co-twins.

Angiopoetin 2B, encoded by the *ANGPT2* gene, is linked to vascular remodelling and the induction of endothelial cell apoptosis. This protein plays a significant role in placental development in humans, ewes, and mice (Dunk et al. 2000; Hess et al. 2006; Zhang et al. 2016). The role of angiopoetin depends on the presence of proangiogenic factors, especially vascular endothelial growth factor (VEGF), whose level is modulated by its soluble receptor (sVEGFR known as sFlt-1) in placentas complicated by IUGR (Khalig et al. 1999; Nevo et al. 2008; Yinon et al. 2014). In the presence of VEGF, angiopoetin enables endothelial cell migration, proliferation and angiogenesis but when VEGF is absent, angiopoetin 2 expression induces vascular regression (Holash et al. 1999; Kappou et al. 2014; Maisonpierre et al. 1997). It has been proposed that angiopoetin 2 inhibition influences the reduction of inflammatory-associated changes in the cells of the endothelium and allows the blood vessels to normalize (Falcon et al. 2009).

**Fig. 3:**
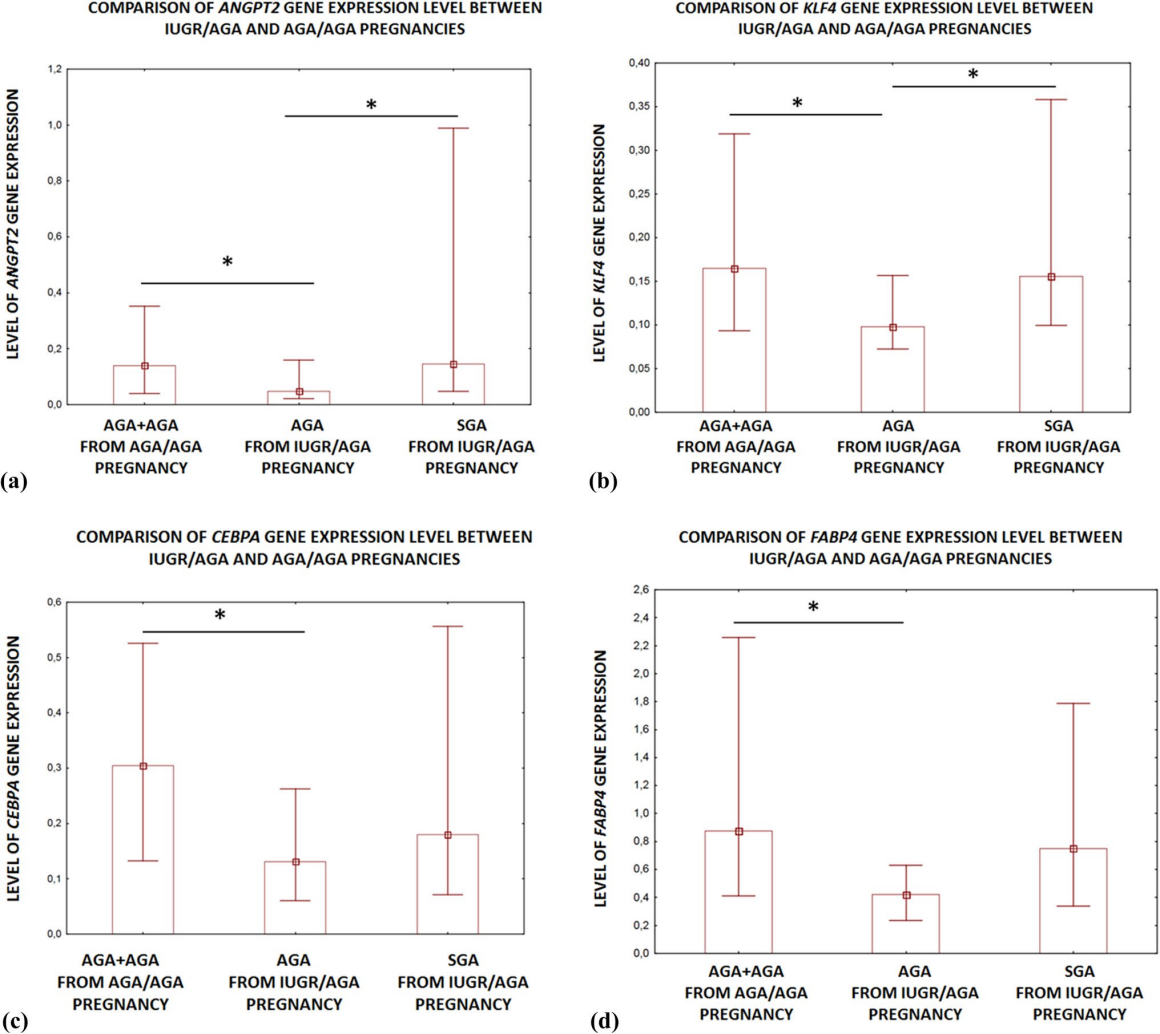
Comparison of *ANGPT2* (**a**), *KLF4* (**b**), *CEBP2* (**c**) and *FABP4* (**d**) expression between AGA from AGA/AGA and SGA and AGA twins from IUGR/AGA pregnancies. Data are presented as median and upper-lower quartile (Mann–Whitney *U* test, *p < 0.01)

Elevated *ANGPT2* expression has been observed in the placentas of singleton preeclamptic pregnancies complicated by IUGR (Kappou et al. 2014). However, a study of gestationally matched placentas from the third trimester of pregnancies (*N* = 10) and from IUGR gestations (*N* = 4), found no significant change in *ANGPT2* mRNA expression (Dunk et al. 2000). In the present study, a higher level of *ANGPT2* gene expression was observed in the placentas of SGA (IUGR) twins compared to their AGA siblings. This might suggest that the upregulation of this gene is associated with the attempt of the small twin to adapt to the local placental environment. However, *ANGPT2* gene was found to be downregulated in the placenta of the AGA twin from the IUGR/AGA pregnancy compared to the two AGA twins in the AGA/AGA pregnancy; whereas, the placentas of SGA (IUGR) fetuses demonstrated similar expression to the AGA fetuses from the AGA/AGA pregnancies (Fig. 3a). It is therefore possible that in case of the AGA fetus from the IUGR pregnancy the *ANGPT2* downregulation (protecting the utero/placental vessels against the regression characteristic for IUGR pregnancies where VEGF is depleted or inhibited by sVEGFR), may be an adaptive mechanism to obtain the proper growth. It is well known that mammalian placentas secrete leptin into maternal blood (Gavrilova et al. 1997). This may be one of the mechanisms by which the maternal metabolism mobilizes fat and provides the fetus with nutrient supplies (Laivuori, et al. 2006). Our findings indicate that leptin expression is higher in the placentas of the SGA (IUGR) twins than in their AGA co-twins. In addition, it is possible that the dysregulation of leptin gene expression between SGA (IUGR) and AGA co-twins may be associated with the local environment typical for the SGA fetus, as indicated by the lack of any significant differences between the leptin gene expression by AGA of IUGR/AGA vs. both AGA co-twins from AGA/AGA pregnancies, and between SGA + AGA (from IUGR/AGA pregnancies) vs AGA + AGA (from AGA/AGA gestations). Numerous studies show that leptin produced by trophoblastic cells is responsible for regulation of placental angiogenesis (Hoggard et al. 2001; Park et al. 2001). It has been proposed that the upregulation of leptin gene expression and its concentration in the placental cells of fetuses born from IUGR pregnancies compensate for any induced growth restriction in the placenta (Schrey et al. 2013). A study conducted on MC twin pregnancies with IUGR found that *LEP* gene expression was significantly higher in the placentas of SGA fetuses than in their co-twins (FC = 24.59, *p* < 0.05), this relationship was also observed on the protein level (Schrey et al. 2013). On the contrary, Laivuori et al. (2006) found that the placental leptin gene expression did not differ between IUGR and uncomplicated pregnancies.

Our present findings indicate that *KLF4* and *GLIS3* genes are dysregulated in the placentas of SGA (IUGR) twins compared to their co-twins, *KLF4* gene is upregulated whereas *GLIS3* is downregulated. Both genes encode the proteins that act as activators or repressors of the transcription process. *KLF4* is mainly engaged in the regulation of normal placentation and apoptosis (Blanchon et al. 2001; Rowland et al. 2005) while *GLIS3* is implicated in the development of some fetal organs, such as the pancreas or thyroid (Dimitri 2017; Dimitri et al. 2011; Nogueira et al. 2013). Some case studies indicate that patients with a *GLIS3* gene mutation resulting in a truncated non-functional protein demonstrated IUGR (Dimitri et al. 2011).In addition, patients with disturbances in the *GLIS3* gene demonstrate impaired beta cell function and an altered fasting glucose and diabetes mellitus phenotype (Wen and Yang 2017; Yang et al. 2013). Further functional studies are needed to establish why the *GLIS3* gene is depleted in the placentas of SGA (IUGR) fetuses developing in a limited energy environment. It is possible that *GLIS3* downregulation might lead to the disturbances in insulin level and in the glucose metabolism of the SGA fetus; these may be intended to prevent significant consumption of glucose by the peripheral tissues and prevent the central hypoglycemia and thus support the brain glucose uptake. This regulation may prevent the death of the SGA fetuses. Numerous studies have noted that SGA fetuses demonstrate a similar level of glucose in the cord blood as AGA fetuses, despite the concentration of insulin being significantly depleted (Åkerman et al. 2018; Bozzetti et al. 1988; Unterman et al. 1993).

The reduced amounts of oxygen and nutrients available to fetuses in pregnancies complicated by IUGR result in enhanced placental apoptosis and aberrant angiogenesis (Arroyo and Winn 2008; Gaccioli and Lager 2016). Kruppel-like factor 4 (KLF4) has been implicated in both processes. Our present findings indicate that *KLF4* gene expression is upregulated in the placentas of SGA (IUGR) fetuses in comparison to their AGA co-twins. However, it was also found that the discrepancy in *KLF4* gene expression between SGA and AGA co-twins (from IUGR/AGA pregnancies) was due to *KLF4* downregulation by the AGA fetus rather than its upregulation by the SGA fetus. SGA (IUGR) fetuses present similar gene expression as AGA fetuses from AGA/AGA pregnancies (Fig. 3b). This finding suggests that *KLF4* downregulation is needed to maintain appropriate fetal weight in a limited energy and oxygen environment. This downregulation has been found to negatively correlate with VEGF in numerous studies (Ray et al. 2013; Tiwari et al. 2013). The human VEGF promoter contains an overlapping site for KLF4, which suppresses VEGF expression and is believed to be a regulator of cell apoptosis. However, the role of KLF4 with regard to apoptosis is dependent on its genetic and cellular context (Malik et al. 2015). Therefore, further studies are warranted to determine the role of KLF4 in placentas of SGA and AGA fetuses born from IUGR/AGA twin pregnancies.

Although no differences were found between the twins born from IUGR/AGA (SGA + AGA) and AGA/AGA (AGA + AGA) pregnancies regarding the expression of *ANGPT2*, *LEP*, *KLF4* and *GLIS3*, the profiles of the *CEBPA* (CCAAT/enhancer-binding protein alpha) and *FABP4* genes differed. Both the genes implicated in the proliferative processes and processes involved in the regulation of liver, adipose tissue, and placenta function (Scifres et al. 2011, 2012; Voutila et al. 2017) were significantly depleted in IUGR pregnancies. In addition, *CEBPA* and *FABP4* gene expression was found to be downregulated in AGA fetuses from IUGR/AGA pregnancies compared to AGA fetuses from AGA/AGA gestations (FC = − 2.1, FC = − 1.7; *p* < 0.01; respectively) (Fig. 3c, d). As these genes were depleted in the total IUGR/AGA group (SGA + AGA) and in the AGA twins from IUGR/AGA pregnancies with regard to AGA/AGA group, and no difference in gene expression was found between SGA (IUGR) and AGA (co-twins), it is possible that this downregulation may be related with unknown maternal factors. These maternal factors may modulate the process of placentation of both twins where one of them (AGA) adapts more efficiently than the other (SGA).

In addition, both maternal and local uterine factors affect the fetal development and may lead to the initiation of the phenomenon called “thrifty genes”. The two-hit hypothesis has been proposed to account for the programming of the health pathology of adult people due to their prenatal insults This hypothesis suggests that a genetic susceptibility combined with an insult occurring during perinatal life (“first-hit”) leads to the reorganization of the various organ systems, which alone might be insufficient to alter the adult phenotype. However, endocrine imbalances resulting from the perinatal insults and postnatal life factors may act as a “second-hit,” which may induce a disease state by unmasking or amplifying the underlying defects (Padmanabhan et al. 2015). It is well documented that the children born from pregnancies complicated by IUGR tend to develop diabetes mellitus, metabolic syndrome or cardiovascular in their postnatal life. Further studies are needed to determine whether the differences observed in the analysed genes of fetuses in the present study may induce differences in children development and their adult metabolism.

The limitation of our study is the fact that DC twins are not genetically identical, as individual fetuses may have different potential to grow and develop IUGR. Paternal factors make them exhibit individual features similar to those siblings who are not twins. This may influence the pace of growth of the fetus and account for some of the variance in our experimental model. However, the lack of any discrepancy in the gene expression between AGA siblings born from AGA/AGA pregnancies suggests that the expression profile of the studied genes does not depend on the genotype of the fetuses but is associated with the unfavourable intrauterine condition. A second limitation is that the present study did not assess the level of proteins encoded by the analysed genes or perform any functional study. Therefore, our future research strategy will comprise conducting laboratory analyses on the protein level and performing functional studies to confirm our suggestion presented in this manuscript.

Conclusions: as both fetuses develop in the same maternal environment, a DC twin pregnancy is a good model to identify differences in gene expression between SGA (IUGR) and AGA co-twins. A difference in gene expression levels between SGA and AGA co-twins could be connected with the adaptation of fetuses to the local uterine environment.
